# Examining the interplay between cardiovascular disease and cancer incidence: Data from NHANES III and continuous

**DOI:** 10.1016/j.ahjo.2024.100380

**Published:** 2024-03-11

**Authors:** Omar M. Makram, Harikrishnan Hyma Kunhiraman, Ryan A. Harris, Catherine C. Hedrick, Khurram Nasir, Neal L. Weintraub, Xiaoling Wang, Avirup Guha

**Affiliations:** a Department of Medicine, Medical College of Georgia at Augusta University, Augusta, GA, USA; b Cardio-Oncology Program, Department of Medicine, Cardiology Division, Medical College of Georgia at Augusta University, Augusta, GA, USA; c Division of Cardiology, Department of Medicine, Medical College of Georgia at Augusta University, Augusta, GA, USA; d Georgia Prevention Institute, Medical College of Georgia at Augusta University, Augusta, GA, USA; e Immunology Center of Georgia, Augusta University, Augusta, GA, USA; f Division of Cardiovascular Prevention and Wellness, Houston Methodist DeBakey Heart and Vascular Center, Houston, TX, USA; g Center for Cardiovascular Computational Health and Precision Medicine (C3-PH), Houston Methodist, Houston, TX, USA

**Keywords:** Cardio-oncology, NHANES, Cancer, CVD

## Abstract

**Introduction:**

This study aimed to investigate the relationship between risk factors of cancer among individuals with existing cardiovascular disease (CVD).

**Methods:**

The analysis included 438 and 2100 CVD patients aged 65+ from NHANES-III and Continuous datasets, respectively. Competing risk models with subdistribution hazards ratio (aHR) were used to identify risk factors.

**Results:**

Females in NHANES-III had lower cancer risk (aHR 0.39, *P* = 0.001) compared to males. Poor physical activity was associated with increased cancer risk in both datasets (aHR 2.59 in NHANES-III, aHR 1.59 in Continuous). In NHANES-Continuous, age (aHR 1.07, *P* < 0.001) and current smoking (aHR 2.55, *P* = 0.001) also showed a significant association with developing cancer. No other factors investigated showed significant associations.

**Discussion:**

This study highlights the interplay between traditional risk factors and the elevated risk of cancer in CVD patients. Further research with larger samples and wider age ranges is needed to solidify these findings and inform intervention strategies.

Several studies have demonstrated that cardiovascular disease (CVD) is a risk factor for cancer development [1,2]. However, among individuals with existing CVD, it remains unclear how traditional cardiovascular risk factors might modify the relationship between CVD and cancer, with limited follow-up in most studies due to the lengthy time-to-event for cancer development. In this study, we aimed to investigate the risk factors of cancer development among CVD patients aged 65+ who participated in the National Health and Nutrition Examination Survey (NHANES) III (1988–1994) or Continuous (1999–2016), were enrolled in Medicare, and without prior cancer. The Medicare mortality data was supplemented with data from the National Death Index (NDI). The earliest death date was utilized if two dates were available. Overall missingness was below 5 %. Diagnosis of CVD was self-identified using the NHANES questions of “Doctor ever told you had: heart failure, heart attack or stroke”. Cancer development was defined as a new cancer diagnosis identified using Medicare part A or B claims or cancer-related death, whichever comes first.

In the final analysis, we included 438 and 2100 patients representing 2,913,888 and 58,194,256 weighted patients with existing CVD from NHANES-III and Continuous, respectively. The data weighting was conducted by the CDC's National Center for Health Statistics (NCHS) and the process included four weighting methods: basic weights (design weights), non-response adjustment, trimming, and poststratification adjustment. Then the final weight for each individual was estimated based on the product of the four measures. Demographics, socioeconomic status, physical activity (PA) level, smoking status, body mass index (BMI), history of diabetes, hypertension, and dyslipidemia, were assessed at the time of participation in both surveys are presented in Table 1. Recommended PA was defined as achieving a minimum of 75 min of vigorous activity or 150 min of moderate-to-vigorous activity per week. Poor PA was characterized by the absence of any recorded physical activity. Intermediate PA involved physical activity falling below the recommended physical activity levels and higher than zero minutes of PA. In the risk factor analysis, we classified smoking into three categories: current, former, and never smokers. Additionally, drinking status was stratified into those who consumed safe daily amount, unsafe alcohol intake, and no drinking.

**Table 1:** General characteristics of participants at baseline.

	NHANES III (1988–1994)	NHANES Continuous (1999–2016)
Non-weighted Sample Size	438	2100
Weighted Sample Size	2,913,888	58,194,256
Age, Years (IQR)	71.9 (68.5–77.0)	73.4 (68.4–79.2)
Female, %	1,603,500 (55.0)	27,304,657 (46.9)
Race/Ethnicity, %		
NH White	2,474,835 (84.9)	46,015,984 (79.1)
NH Black	267,777 (9.2)	5,356,029 (9.2)
Hispanic	73,006 (2.5)	1,845,494 (3.2)
Other Race	98,270 (3.4)	5,006,749 (8.6)
Socioeconomic Status		
Education levels, %*		
<9th Grade	801,676 (27.5)	8,322,403 (14.3)
9–11th Grade	513,809 (17.6)	9,426,474 (16.2)
High School/GED	917,044 (31.5)	16,397,733 (28.2)
Some College or AA Degree	681,359 (23.4)	14,462,847 (24.9)
College Graduate or Above	–	9,493,977 (16.3)
Marital Status, % of Married	1,627,632 (55.9)	32,394,754 (55.7)
Family Income/Poverty Ratio (IQR)	1.9 (1.3–3.2)	2.2 (1.3–3.7)
Access to Healthcare, % of Yes	2,750,567 (94.4)	57,216,343 (98.3)
Cardiovascular Risk Factors		
Diabetes, % of Yes	530,474 (18.2)	17,727,840 (30.5)
Dyslipidemia, % of Yes	1,233,715 (42.3)	36,793,758 (63.2)
Hypertension, % of Yes	1,709,285 (58.7)	42,288,125 (72.7)
Current Smokers, %	289,581 (9.9)	6,617,584 (11.4)
Physical Activity Levels, %		
Recommended	1,141,828 (39.2)	12,271,608 (21.1)
Intermediate	888,290 (30.5)	9,193,599 (15.7)
Poor	883,769 (30.3)	33,074,441 (56.8)
BMI, kg/m^2^ (IQR)	26.6 (23.8–30.7)	28.4 (25.1–32.7)

AA: Associate of Arts; BMI: Body mass index; GED: General education development; IQR: Inter-quartile range; kg: Kilogram; m^2^: Meter square; NH: Non-Hispanic; %: Percentage; *missing % <10 % but used in tabulation so field does not total 100 %.

The risk factor analysis included all the variables listed in Table 1. We utilized the SURVEYPHREG methodology in SAS 9.4, which accounted for NHANES sampling year-appropriate cluster, strata, and sample weights after eliminating empty clusters. In our modeling for risk factor analysis, we used an a-priori set of variables as following: age, sex, race/ethnicity socioeconomic status, smoking, PA, alcohol use, BMI, history of hypertension, diabetes, and dyslipidemia. Competing risk of non-cancer mortality was utilized to generate an adjusted subdistribution Hazards ratio (aHR) with a 95 % confidence interval (95%CI). We further conducted an interaction test; however, we observed no effect modification among the included variables in Table 1 in either dataset.

NHANES-III participants had a median age of 71.9 years (interquartile range = 68.5–77.0), 55 % were females, 84.9 % were self-identified as non-Hispanic White individuals, and 58.7 % had hypertension. In NHANES-Continuous group, participants were slightly older with a median age of 73.4 years, with fewer females (46.9 %) and non-Hispanic Whites (79.1 %), but with higher prevalence of hypertension (72.7 %). The median follow-up for NHANES-III and Continuous were 12.2 and 6.7 years, respectively. Over that period, in NHANES-III, breast cancer, colorectal cancer, prostate cancer, and lung cancer were newly diagnosed in 1 %, 0.5 %, 5.7 % and 3.3 % of patients, respectively; with crude cancer death rate of 9.4 % (Fig. 1). In the competing risk models, among patients with CVD, females had a lower risk of developing cancer than males (aHR 0.39; 95%CI 0.22–0.68; *P* = 0.001), while poor PA was associated with a higher risk of cancer development compared to recommended PA levels (aHR 2.59; 95%CI 1.11–6.04; *P* = 0.03).

**Fig. 1:**
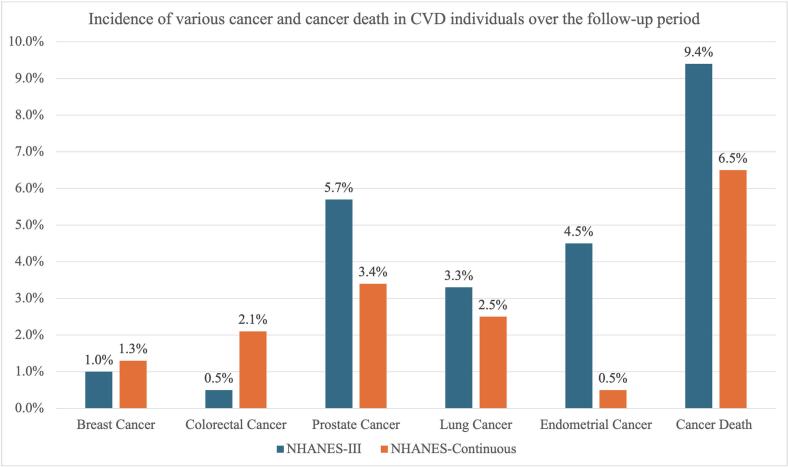
Incidence of various cancers and cancer death in CVD individuals.

In NHANES-Continuous, a similar pattern was found, with 1.3 %, 2.1 %, 3.4 %, 2.5 % of patients newly diagnosed with breast cancer, colorectal cancer, prostate cancer, and lung cancer, respectively, although the rate of cancer death was lower (6.5 % vs 9.4 %) (Fig. 1). Additionally, although sex did not play a significant role as a risk factor for cancer in this survey, age did (aHR 1.07; 95%CI 1.04–1.10; *P* < 0.01). Additionally, current smokers had higher risk of cancer, compared to never smokers (aHR 2.55; 95%CI 1.46–4.46; *P* = 0.001); and those with poor PA levels had higher risk compared to recommended PA level (aHR 1.59; 95%CI 1.05–2.41; *P* = 0.03). None of the other variables presented in Table 1 showed a significant association with increased risk of cancer in either survey.

Findings from the present investigation are consistent with those of Lau et al., who demonstrated a higher risk of cancer in those with elevated ASCVD risk score [3]. Also, similar findings were found in a sample of Japanese population with CVD over a period of 3 years [4] and in post-menopausal women with heart failure from the Women's Health Initiative [2]. It is important to note, however, that the prior results were demonstrated in patients with either no prior CVD, non-US population, short follow-up, or only in heart failure patients. Lastly, consistent with our results, a previous systemic review concluded that higher levels of physical activity are not only linked to reduced risk of cancer development, but also improved survival in different types of cancer [5].

In conclusion, the present study highlights the interplay between traditional cardiovascular risk factors and cancer development in those with existent CVD. Nevertheless, this study is limited by the sample size and the representativeness of the included subset of patients from NHANES datasets, particularly with respect to race and ethnicity. Additional studies with larger sample sizes, a wider age range, and more representation for Hispanics and Non-Hispanic Blacks are needed to further understand the dynamics of the complex interaction between cancer and CVD and its implications for prevention and intervention strategies.

## Source of funding

This project received funding from the 10.13039/100000968American Heart Association (AHA) Strategically Focused-Research Network (SFRN) on disparities in Cardio-Oncology.

## Disclaimer

The findings and conclusions in this paper are those of the author(s) and do not necessarily represent the views of the Research Data Center, the National Center for Health Statistics, or the Centers for Disease Control and Prevention.

## CRediT authorship contribution statement

**Omar M. Makram:** Writing – review & editing, Writing – original draft. **Harikrishnan Hyma Kunhiraman:** Writing – review & editing. **Ryan A. Harris:** Writing – review & editing. **Catherine C. Hedrick:** Writing – review & editing. **Khurram Nasir:** Writing – review & editing. **Neal L. Weintraub:** Writing – review & editing, Supervision. **Xiaoling Wang:** Writing – review & editing. **Avirup Guha:** Writing – review & editing, Writing – original draft, Supervision, Methodology, Formal analysis.

## Declaration of competing interest

The authors declare the following financial interests/personal relationships which may be considered as potential competing interests:

Avirup Guha reports financial support was provided by the 10.13039/100000968American Heart Association. If there are other authors, they declare that they have no known competing financial interests or personal relationships that could have appeared to influence the work reported in this paper.
